# Effect of the knee replacement surgery on activity level based on ActivPAL: a systematic review and meta-analysis study

**DOI:** 10.1186/s12891-022-05531-2

**Published:** 2022-06-15

**Authors:** Huda Alfatafta, Mahmoud Alfatafta, David Onchonga, Sahar Hammoud, Haitham Khatatbeh, Lu Zhang, Imre Boncz, Szimonetta Lohner, Bálint Molics

**Affiliations:** 1 Doctoral School of Health Sciences, Faculty of Health Sciences, University of Pécs, 7621, Vörösmartyutca 4, Pécs, Hungary; 2 Orthotics and Prosthetics Department, Rehabilitation Sciences School, University of Jordan, Amman, Jordan; 3 Institute for Health Insurance, Faculty of Health Sciences, University of Pecs, Pécs, Hungary; 4 Cochrane Hungary, Clinical Center, Medical School, University of Pécs, Pécs, Hungary; 5 Institute of Physiotherapy and Sport Science, Faculty of Health Sciences, University of Pecs, Pécs, Hungary

**Keywords:** Knee replacement, ActivPAL, Activity level

## Abstract

**Background:**

The knee replacement (KR) surgery aims to restore the activity level and reduce the risk of experiencing disabilities. The outcomes of this surgery are evaluated mainly with subjective tools or low validity objective tools. However, the effect of the surgery on activity level using high validity objective accelerometer is still in question.

**Methods:**

A systematic review and meta-analysis were conducted to evaluate the benefit of KR surgery alone to enhance physical activity recommendations based on high validity accelerometer. Two independent reviewers evaluated five electronic databases (Cochrane-Central-Register-of-Controlled Trials, EMBASE, PubMed, Web of Science, and Scopus) to find relative studies between January 2000 and October 2021. The quality assessments and risk of bias assessments were examined.

**Results:**

Three articles were included with 202 participants (86 males, 116 females), with an average age of 64 years and an average 32 kg/m^2^ body mass index. The results found that the number of steps was significantly improved up to 36.35 and 45.5% after 6-months and 1-year of the surgery, respectively. However, these changes did not meet the recommended activity level guideline and could be related to the patients’ health status and their activity level before the surgery. No significant changes were seen in sedentary time, standing time, and upright time after 6-months and 1-year follow-ups. Heterogeneity among studies was low to moderate (0–63%).

**Conclusion:**

Knee replacement surgery is an effective treatment for improving patients’ quality of life with severe knee injuries. However, various factors impact the success of surgical and achieving maximum benefit of the surgery. One factor, sedentary time, can be reduced by implementing pre-and post-surgery exercise or physical activity recommendations. Further studies are needed to understand the benefit of surgery with or without rehabilitation assessed using high validity monitors.

**Supplementary Information:**

The online version contains supplementary material available at 10.1186/s12891-022-05531-2.

## Background

Knee replacement surgery (KR) is the last surgical intervention to deal with severe knee injuries such as advanced knee osteoarthritis [1, 2]. The main outcomes of this surgery are reducing the pain and increasing the quality of life and the physical activity (PA) level of the patients [2]. The success of this surgery depends on the patients’ self-satisfaction in terms of quality of life improvement after the surgery including the physical improvement [2, 3]. Physical improvement is not only important to increase self-satisfaction but also to enhance musculoskeletal and cardio-respiratory functions, reduce the risk of falls, improve physical function, and reduce the risk of death [4].

Most of the available studies that evaluated the PA level after the surgery used subjective methods only such as questionnaires [5–7]. The mainly used questionnaires that evaluate the quality of life and the PA level improvements are the 36-item Short-Form health survey (SF-36), the Western Ontario and McMaster Universities Osteoarthritis Index (WOMAC), the Knee injury and Osteoarthritis Outcome Score (KOOS), and the Oxford Knee Score (OKS) [8]. However, those questionnaires are subjective evaluation methods and are associated with limited reliability and recall bias [8, 9]. Therefore, PA after knee OA surgery evaluated with precise methods such as ActivPAL remains unclear.

Several studies have relied on different types of objective monitors (accelerometer or pedometer); however, most of these monitors have low validity and reliability [10–12]. On the other hand, few studies have used high validity and reliability objective monitors to measure PA level among the elderly population. To our knowledge, no systematic review and meta-analysis studies focused on evaluating the PA level after the surgery based on only high validity and reliability objective monitors such as ActivPAL (PAL Technologies, Glasgow, UK).

ActivPAL is a lightweight (20 g) subjective uniaxial accelerometer that is used widely to evaluate the PA level. This monitor detects the inclination of the thigh to determine body movement [13–16]. The ActivPAL is a valid and reliable device to measure the time spent in sedentary, standing, upright and stepping states and the number of steps per day. The reliability of the ActivPAL is considerably high (between 0.97–0.99) [14, 15]. It is valid to evaluate children, adults, and the elderly. Additionally, it is valid to assess the slow walking population with less than 1% absolute misclassification error [14, 15, 17–19]. Compared with other accelerometers, using hip/thigh-worn accelerometers and wrist-worn accelerometers cannot distinguish between walking and stair climbing activities; besides, they cannot distinguish between sitting and lying down positions [19]. Therefore, the ActivPAL is more recommended to be used with the elderly population than other monitors to evaluate slow walking and distinguish between different activities and postures [19, 20]. For the previously mentioned criteria of the ActivPAL, this study focused on evaluating the studies that used the ActivPAL as a monitor for data collection.

The patients who decided to make the KR surgery are expecting to reach the outcomes of the surgery. However, the outcomes of the surgery are still doubtful as some patients feel that their activity level after the surgery did not change significantly, while only less than 5% of them had restored their activity level after 1–2 years of the surgery [21, 22]. Additionally, their activity level after the surgery still does not meet the recommended guidelines of the activity level of 150 minutes per week of moderate-intensity physical activities [23, 24]. Thus, it is critical to identify the activity level enhancement after the KR surgery using high validity monitor. To date, no systematic review is available to determine the PA level improvement using the ActivPAL. Hence, this study aims to understand the objective improvement after the KR surgery to find out if this surgery could significantly enhance the quality of life or not, based on a high-quality accelerometer.

## Methods

This meta-analysis study is reported based on the Preferred Reporting Items for Systematic Reviews and Meta-analysis (PRISMA) guidelines [25] (Additional file 1: Appendix 1).

### Search strategy

Five electronic databases including Cochrane Central Register of Controlled Trials, EMBASE, PubMed, Web of Science, and Scopus were searched for relevant studies. Two independent reviewers conducted search based on the search strategy. This strategy was adapted for the different databases as required (Additional file 1: Appendix 2). The search was performed from January 2000 until the end of October 2021.

### Study screening

Two authors independently selected studies based on predefined inclusion criteria. The titles and abstracts were reviewed first, and irrelevant references were excluded. Then, the reviewers screened the full-text publications of potentially relevant studies. The references and related articles of the selected studies were screened for more suitable studies. Any disagreement was resolved by discussion among the two authors with the possibility to involve a third author as a consultant to make a final decision. Authors were contacted for more information or clarifications if needed.

### Eligibility criteria

All English language published studies that evaluated the PA level improvement before and after the knee replacement surgery using the ActivPAL included regardless of the study designs. Moreover, the included articles must meet the following criteria: (a) adult participants with severe knee OA who received the KR surgery, (b) minimum follow-up time is 6 months, and (c) the PA level is measured by the ActivPAL only. The study was excluded if (a) it combined the knee replacement surgery with any other interventions, or (b) used another accelerometer.

### Data extraction and risk-of-bias assessment

The two reviewers used the same data extracted sheet to report the following aspects: study information (author, year), study design, number of participants, patients ‘demographic, preoperative activity level, postoperative activity level, main findings, and funding resources.

The reviewers evaluated the quality of reporting according to the Strengthening the Reporting of Observational Studies in Epidemiology (STROBE) tool for the non-randomized controlled studies which have 22 items to assess the reporting quality of title and abstract, introduction, methods, results, and discussion sections [26, 27]. Moreover, the Risk Of Bias In Non-randomized Studies (ROBINS-I) tool was used to evaluate the risk of bias in non-randomized controlled studies by evaluating seven dominates of bias (confounding, selection, measurement of interventions, missing data, measurement of outcomes, and reporting) [28]. For the non-randomized uncontrolled studies, the National Institutes of Health (NIH) quality assessment tool was used to evaluate the quality of pre-post studies without a control group [29].

### Statistical analysis

The Cochrane Collaboration’s Review Manager Program (RevMan version 5.3, Cochrane collaboration, Oxford, UK) was used for data analysis. Weighted mean differences (WMDs) and corresponding 95% confidence intervals (CIs) were estimated by a Fixed-effect meta-analysis. The random-effect meta-analysis was used if heterogeneity is more than 50%. The chi-square test for Q and the I^2^ quantity were used to test heterogeneity between studies. Significant results were considered if a *p*-value for the chi-square test was ≤0.1 and I^2^ ≥ 50% [25].

## Results

**Fig. 1:**
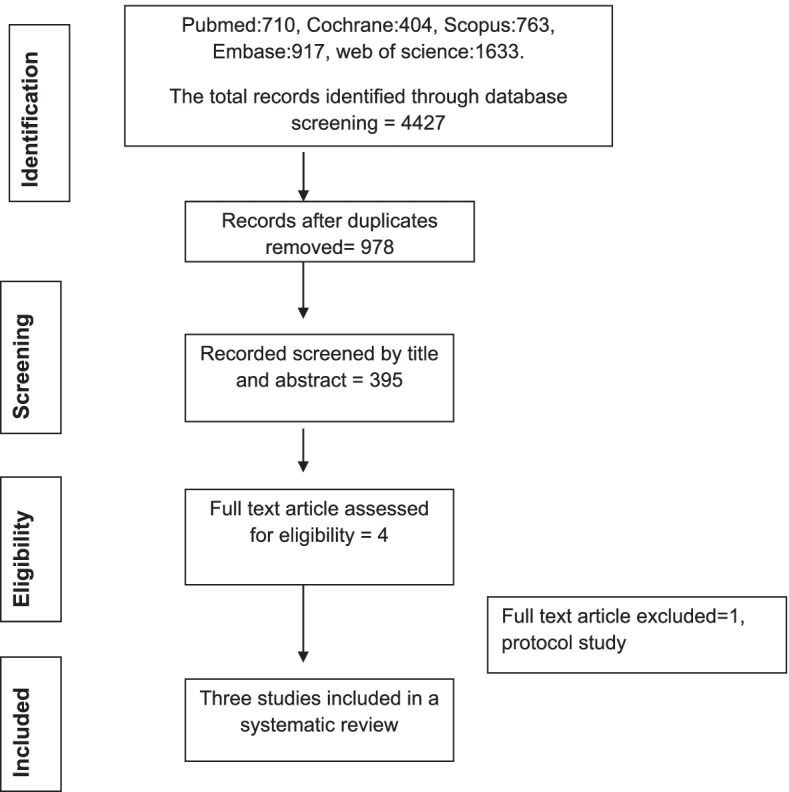
PRIMSA flow chart of the study identification

A total of 4427 relevant studies were found initially. After removing duplicated articles and reviewing the title and the abstract, 395 articles remained. Then, four articles met the inclusion criteria after the full-text examination [23, 30–32] (Fig. 1). Later, one of them was excluded because it was only a protocol study [30]. Finally, three studies were included (two of them were uncontrolled studies). From forward citation searches, 71 articles were assessed, but none of them met the inclusion criteria.

### Systematic review

The three included studies were prospective and only one of them included a control group (Tables 1 and 2). The total number of patients was 173 participants (more than 50% females) with an average age of 63.3 years and an average of 33.2 kg/m^2^ body mass index (Table 1).

**Table 1:** Summary of the included studies. M: male. F: female

Authors	Type of study	Follow up	Number of participants (M/F)	Average age (years)	Average BMI (kg/m^2^)
Granat et al., 2020 [23]	Uncontrolled before-after study	6 months and 12 months	33 (6 m, 27f)	59 ± 6	37.21 ± 7.65 for females, 32.38 ± 2.01 for males
Lützner et al., 2014 [31]	Controlled before-after study	12 months	97 (52 m, 45f)	68.9	31.3 (30.3–32.3)
Frimpong et al., 2020 [32]	Uncontrolled before-after study	6 months	43 (NA)	62.8 ± 8.6	33.8 (±7.1)

**Table 2:**
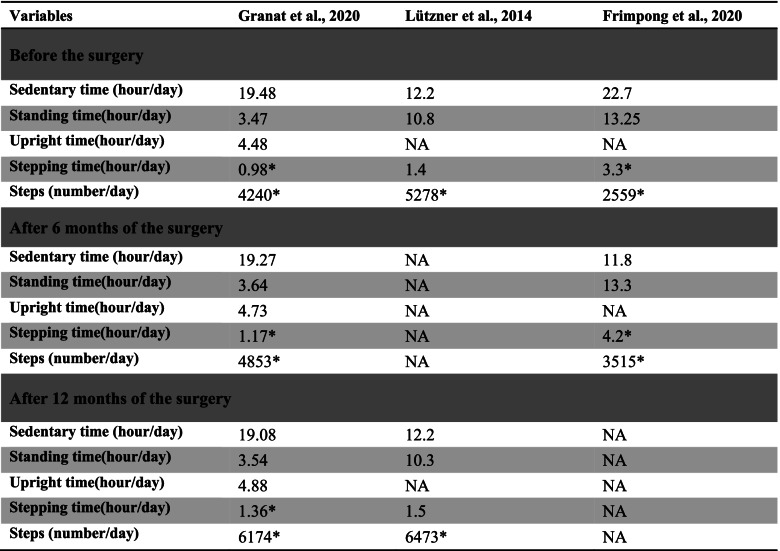
Summary of results of the included studies. The highlighted results are with significant changes. (NA = not available)

Granat et al., [23] evaluated 33 participants after 6-months and 1 year of unilateral total knee replacement surgery. The patients used the ActivPAL for seven consecutive days at each stage. The monitor was attached over the mid-thigh. The results found that the stepping time and steps’ number after 6 months and 1 year of having the surgery significantly improved compared to pre-surgery. The number of steps significantly improved by 14.4 and 45.6% after 6-month and 1 year, respectively. Moreover, the stepping time significantly improved by 11.48 ± 2.05 (19.38%) min/day and by 22.66 ± 2.24 (38.77%) min/day after 6-months and 1 year, respectively. However, the changes in stepping time and the number of steps did not meet the PA guideline of 150 minutes of activity per week. Whilst no significant differences were seen in sedentary time, standing time, and upright time.

Lützner et al., [31] assessed 97 patients after 1 year of unilateral total knee replacement surgery. The patients used the ActivPAL for four consecutive days. The monitor was attached over the anterolateral tibia. This study found that the number of steps increased from 5278 ± 2999 to 6473 ± 3654 steps/day (20.36%) after 1 year of the surgery. However, no significant changes in sedentary, stepping, and standing times were demonstrated. Furthermore, only 16 participants met the PA guidelines.

Frimpong et al., [32] examined 43 participants after 6-months of unilateral total knee replacement surgery. The patients used the ActivPAL for seven consecutive days. The monitor was attached over mid-thigh. The results found that the number of steps significantly improved (with an average of 2559 to 3515 steps/day, *P* = 0.001, 37.35%) and the walking time significantly increased (with an average of 79 to 101 minutes/day, *P* = 0.006, 27.2%) after 6-months of the surgery. Nevertheless, no significant changes in sedentary and sitting times were reported.

### Meta-analysis results

The meta-analysis was used to evaluate the activity level enhancement after 6 months and 1 year. The results revealed that the heterogeneity of the activity level after 6 months and 1 year is low to moderate (Tables 3, 4 and 5). After 6 months of the surgery, the number of steps (2 studies, 164 participants) improved (95% CI = 0.43 (0.11–0.76); *P* = 0.38; I^2^ = 0%) with small heterogeneity. Based on the same two studies, the sedentary time, stepping time, and standing time improved but insignificantly (Table 3). After 1 year of the surgery, the number of steps (three studies, 153 participants) enhanced (95% CI = -0.21 (− 0.36–0.06), *P* = 0.79; I^2^ = 0%) with moderate heterogeneity (Table 3). The sedentary time, stepping time, standing time (two studies, 130 participants) also insignificantly improved after 6 months (95% CI = − 0.22 (− 0.37–0.07), *P* = 0.10; I^2^ = 63) and 1 year (95% CI = 0.47 (0.22–0.71), *P* = 0.11; I^2^ = 45%) (Table 5). So, the overall heterogeneity after 6 months and 1 year was low and up to I^2^ = 45%, P = 0.11 (Tables 3, 4 and 5).

**Table 3:**
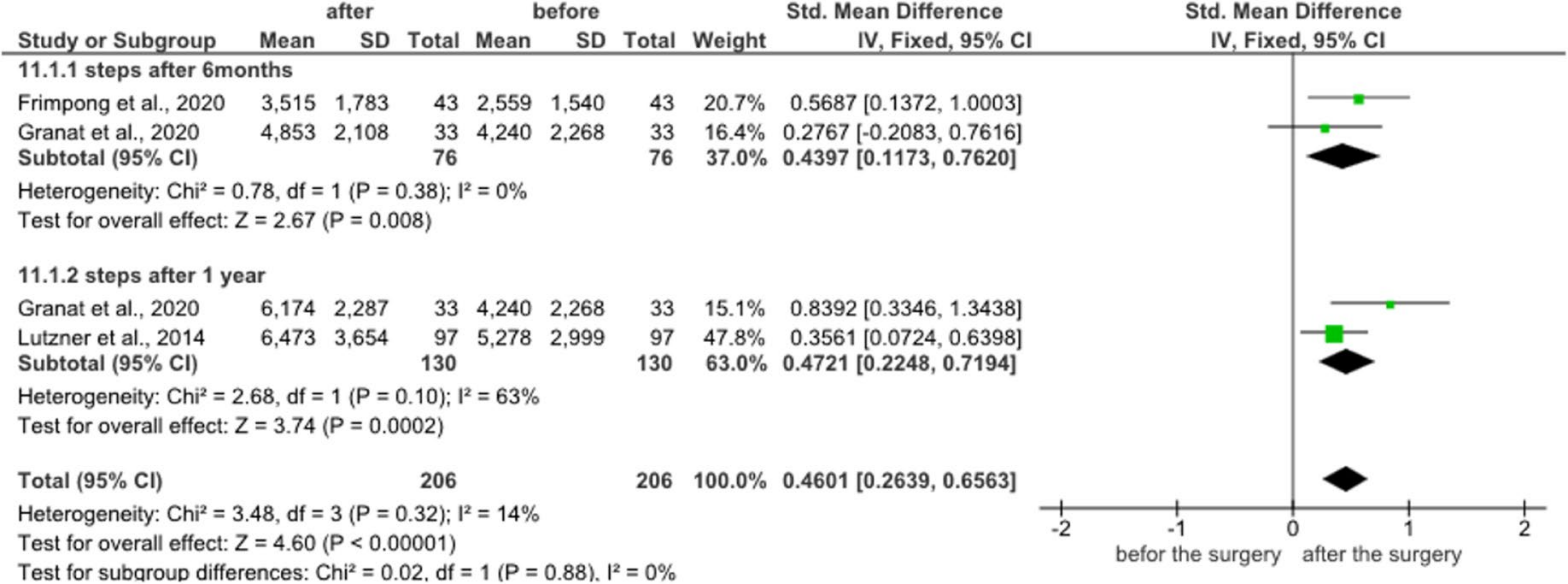
Meta-analysis of number of steps (average number/day) after 6 months and 1 year of the surgery

**Table 4:**
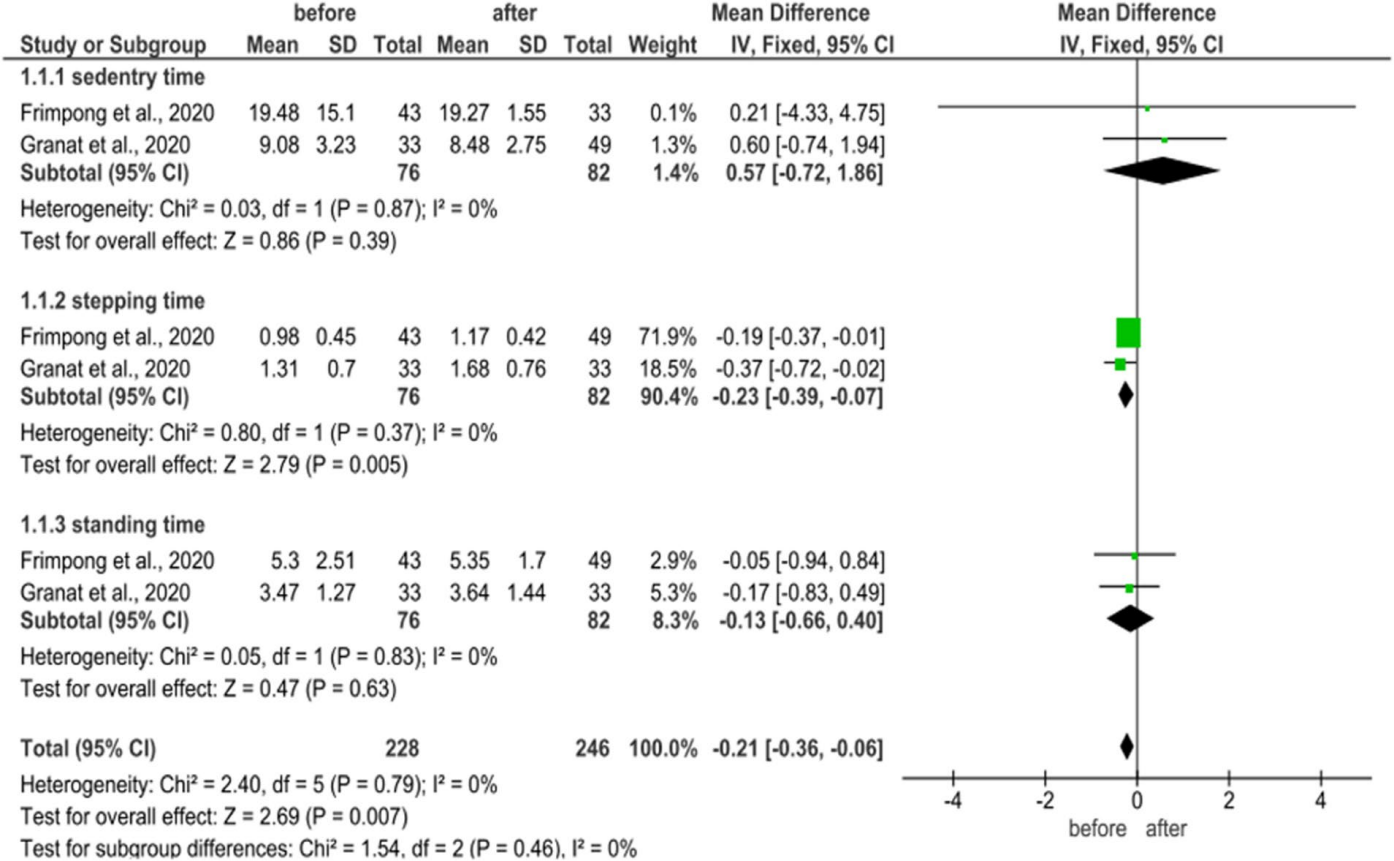
Meta-analysis results of sedentry time, stepping time, and standing time (hour/day) after 6 months of the surgery

**Table 5:**
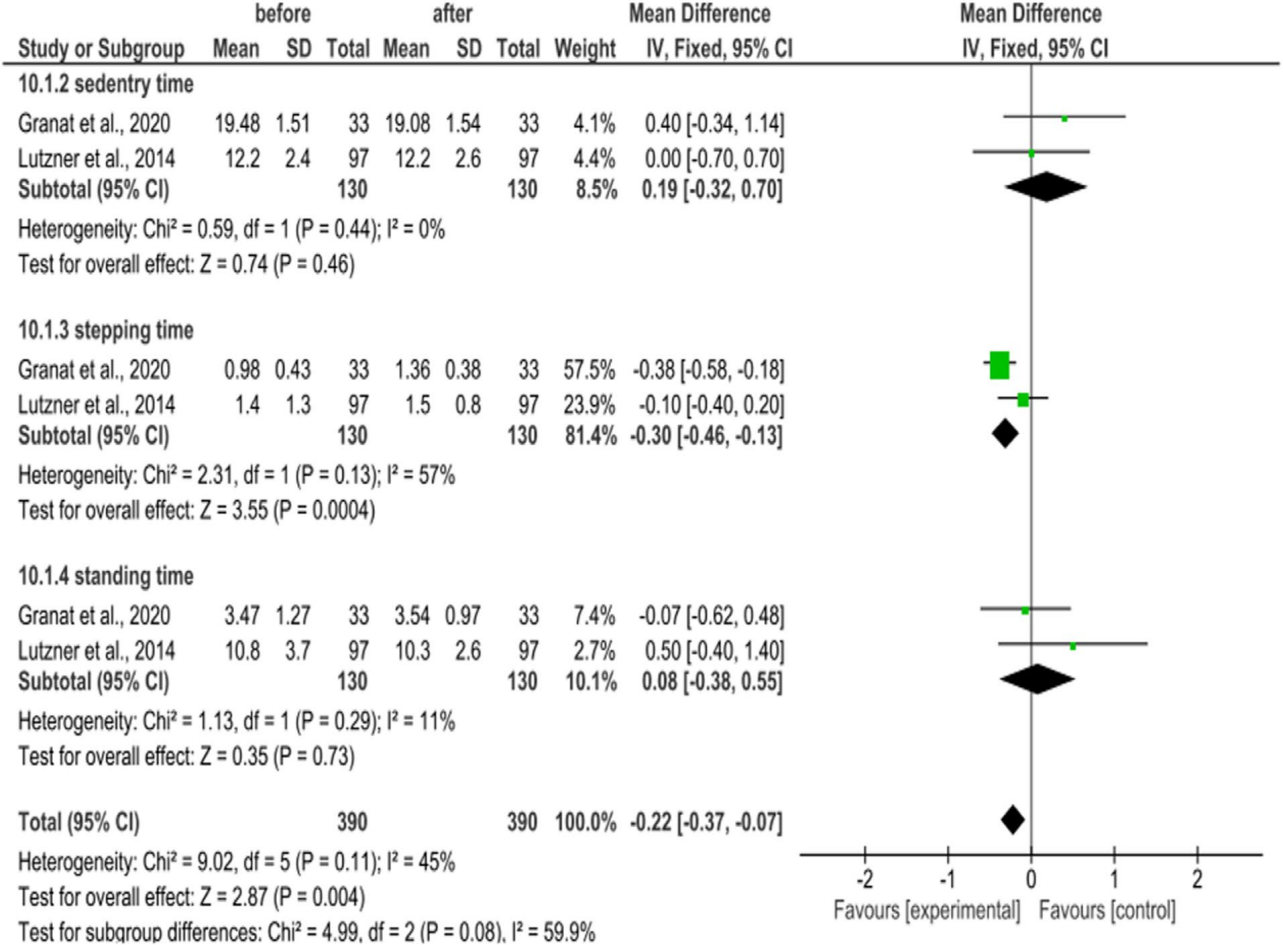
Meta-analysis of sedentry time, stepping time, and standing time (hour/day) after 1 year of the surgery

### Reporting quality and risk of bias assessments

The accepted articles were non-randomized studies; thus, the STROBE tool was used to assess the study’s generalizability. In terms of title and abstract, the three accepted studies had a clear informative abstract. For the introduction, also all the studies provided critical background with specified objectives. In terms of methods and results, all of them clearly described the study design, data collection, recruitment process, participants’ criteria, the main measurable variables, and the main outcomes. For the discussion, all studies revealed the main key points, compared their findings with other studies, and stated the associated limitations. For funding, two studies reported their source of funding [23, 31].

The ROBINS-I tool was used to evaluate the risk of bias in the controlled study, Lützner et al., [31] (Additional file 1: Appendix 3). That study was associated with selection bias and performance bias. The researchers attached the ActivPAL on the tibia which is not a recommended location; besides, it collected the data for 4 days which is not very enough to evaluate the AP. These findings might reduce the generalization of the results.

For studies without a control group, Lützner et al., [31] and Frimpong et al., [32], the NIH quality assessment was used to evaluate the quality and risk of bias (Additional file 1: Appendix 4). The risk of biases, that are associated with the two studies, were selection bias, unblinded participants, and the statistical analysis did not take into account the use of individual-level data to determine effects at the group level. These findings might reduce the quality of the results, and reduce the internal validity.

## Discussion

Knee replacement is not an easy decision-making intervention to cope with severe knee injuries. The patients expect that the KR surgery will help them to restore their physical activity, be more active, and to be more independent. Hence, a systematic review study was conducted to evaluate the PA improvement after the surgery using the ActivPAL. This study focused on the PA that was examined with the ActivPAL as it is a very accurate monitor, suitable to evaluate the sedentary time of the elderly population, and more recommended to be used than the ActiGraph [20]. The main finding of this study is only the number of steps was significantly improved after the surgery among most of the patients [23, 30–32]. Nevertheless, this improvement is still not enough to restore their normal activity level as it did not reach to recommended activity level guideline. Moreover, the sedentary time did not significantly reduce after the KR surgery which might decrease the benefits of the surgery.

It is expected that the outcomes of the included studies are associated with participants’ health status before the surgery. For instance, the average age of the included participants in Granat et al., [23] study was considerably low (59 ± 6 years old, range: 49–76 years old). In Lützner et al., [31] study, the participants had a high number of steps (with an average of 5000 steps/day) before the surgery. Similarly, Frimpong et al., [32] study found significant differences in the number of steps after 6 months and that could be related to including patients with body mass index less than 30 kg/m^2^. Therefore, the age, body mass index and activity level of the patients before the surgery could be correlated with the outcomes of the KR surgery.

Other factors also could have an impact on the outcomes of the KR surgery. It has been suggested that the KR surgery could increase the movement-related activity and number of sit-to-stand movements by 0.7 and 9.7% respectively after 6-months and that depends on the body mass of the patients and the physical treatment after the surgery [33]. Another study found that the male and young age (< 65 years old) patients show better PA levels after the surgery than women and elderly participants [34]. Furthermore, the emotional state of the patients and their partners has an influence on the PA recovery after the surgery [35]. Therefore, further research is required to understand the impact of these factors and find other factors.

Our results match with other studies which evaluated the PA level after at least 6 months of having the KR surgery using other types of activity monitors. These studies also found small changes in the AP after 6 months of the surgery as patients are still inactive and have high sedentary time after the surgery [33–37]. Similarly, the available systematic review studies that reported the PA after the KR surgery using other types of activity monitors found that the changes in the AP after 6-month of the surgery, and only moderate changes could be seen in the PA after 1 year of the surgery but still insufficient [10–12].

To sum up, even the subjective measures such as pain, function, and stiffness might improve after the surgery, not all aspects of the activity level based on the objective tools significantly increased. So far, not enough evidence about the benefit of KR surgery for severe knee OA on the PA level using Activpal is available. Hence, better physical capability after the surgery does not mean a better PA level.

### The limitations

This study is engaged with limitations. Few studies met the inclusion criteria and none of them is a randomized controlled study; therefore, the results of the included studies could be associated with a high risk of bias such as selection bias and performance bias. Also, this study included only studies that used the ActivPAL and excluded studies with any other interventions with the surgery which limits the results’ generalizability.

## Conclusions

Total knee replacement surgery is an effective treatment to improve the quality of life among patients with severe knee injuries. Based on the high validity monitor, the number of steps significantly improved, but the sedentary time did not change. To increase the maximum benefits of the surgery, the sedentary time should be decreased. Hence, long-term follow-ups, rehabilitation programs, and physical interventions are important to enhance the physical outcomes and reduce the sedentary time after the surgery. This finding could be important for specialists who work with the KR patients to restore their activity level after the surgery and make them more satisfied by implementing activities that help them to reduce their sedentary time. The patients’ expectations after the surgery should be discussed with the patients before the surgery.

## Supplementary Information

**Additional file 1.**

## Data Availability

The datasets generated and/or analyzed during the current study are not publicly available because the dataset is very large and to avoid data misuse but are available from the corresponding author upon reasonable request.

## Abbreviations

KR: The knee replacement
PA: Physical activity
SF-36: 36-item Short-form
WOMAC: Western Ontario and McMaster Universities Osteoarthritis Index
KOOS: Knee injury and Osteoarthritis Outcome Score
OKS: Oxford Knee Score
STROBE: Strengthening the Reporting of Observational Studies in Epidemiology tool
ROBINS-I: Risk Of Bias In Non-randomized Studies
NIH: National Institutes of Health
WMDs: Weighted mean differences
CIs: Confidence intervals
