# Supplementary material for: Effect of the knee replacement surgery on activity level based on ActivPAL: a systematic review and meta-analysis study
Source: BMC Musculoskelet Disord. 2022 Jun 15;23:576. doi: 10.1186/s12891-022-05531-2 (PMC9199222; doi:10.1186/s12891-022-05531-2)
Supplement: Supplementary file 1 — Additional file 1. [file 12891_2022_5531_MOESM1_ESM.doc]

| **Section/topic** | **#** | **Checklist item** | **Reported on page #** |
| --- | --- | --- | --- |
| **TITLE** | | |  |
| Title | 1 | Identify the report as a systematic review, meta-analysis, or both. | 1 (line 1) |
| **ABSTRACT** | | |  |
| Structured summary | 2 | Provide a structured summary including, as applicable: background; objectives; data sources; study eligibility criteria, participants, and interventions; study appraisal and synthesis methods; results; limitations; conclusions and implications of key findings; systematic review registration number. | 2 (line 19) |
| **INTRODUCTION** | | |  |
| Rationale | 3 | Describe the rationale for the review in the context of what is already known. | 5 |
| Objectives | 4 | Provide an explicit statement of questions being addressed with reference to participants, interventions, comparisons, outcomes, and study design (PICOS). | 5-6 |
| **METHODS** | | |  |
| Protocol and registration | 5 | Indicate if a review protocol exists, if and where it can be accessed (e.g., Web address), and, if available, provide registration information including registration number. | 7 |
| Eligibility criteria | 6 | Specify study characteristics (e.g., PICOS, length of follow-up) and report characteristics (e.g., years considered, language, publication status) used as criteria for eligibility, giving rationale. | 7 |
| Information sources | 7 | Describe all information sources (e.g., databases with dates of coverage, contact with study authors to identify additional studies) in the search and date last searched. | 8 |
| Search | 8 | Present full electronic search strategy for at least one database, including any limits used, such that it could be repeated. | Appendix 1 |
| Study selection | 9 | State the process for selecting studies (i.e., screening, eligibility, included in systematic review, and, if applicable, included in the meta-analysis). | 7-8 |
| Data collection process | 10 | Describe method of data extraction from reports (e.g., piloted forms, independently, in duplicate) and any processes for obtaining and confirming data from investigators. | 8 |
| Data items | 11 | List and define all variables for which data were sought (e.g., PICOS, funding sources) and any assumptions and simplifications made. | 8 |
| Risk of bias in individual studies | 12 | Describe methods used for assessing risk of bias of individual studies (including specification of whether this was done at the study or outcome level), and how this information is to be used in any data synthesis. | 8 |
| Summary measures | 13 | State the principal summary measures (e.g., risk ratio, difference in means). | 9 |
| Synthesis of results | 14 | Describe the methods of handling data and combining results of studies, if done, including measures of consistency (e.g., I^2^) for each meta-analysis. | 9 |

**Appendix 1: PRISMA check list**

| **Section/topic** | **#** | **Checklist item** | **Reported on page #** |
| --- | --- | --- | --- |
| Risk of bias across studies | 15 | Specify any assessment of risk of bias that may affect the cumulative evidence (e.g., publication bias, selective reporting within studies). | 11 and 12,and Appendix 3 and 4 |
| Additional analyses | 16 | Describe methods of additional analyses (e.g., sensitivity or subgroup analyses, meta-regression), if done, indicating which were pre-specified. | N/A |
| **RESULTS** | | |  |
| Study selection | 17 | Give numbers of studies screened, assessed for eligibility, and included in the review, with reasons for exclusions at each stage, ideally with a flow diagram. | 9 |
| Study characteristics | 18 | For each study, present characteristics for which data were extracted (e.g., study size, PICOS, follow-up period) and provide the citations. | 9 and tables 1and 2 |
| Risk of bias within studies | 19 | Present data on risk of bias of each study and, if available, any outcome level assessment (see item 12). | 11,and Appendix 3 and 4 |
| Results of individual studies | 20 | For all outcomes considered (benefits or harms), present, for each study: (a) simple summary data for each intervention group (b) effect estimates and confidence intervals, ideally with a forest plot. | 11 and Tables3-5 |
| Synthesis of results | 21 | Present results of each meta-analysis done, including confidence intervals and measures of consistency. | Tables 3-5 |
| Risk of bias across studies | 22 | Present results of any assessment of risk of bias across studies (see Item 15). | 11,and Appendix 3 and 4 |
| Additional analysis | 23 | Give results of additional analyses, if done (e.g., sensitivity or subgroup analyses, meta-regression [see Item 16]). | N/A |
| **DISCUSSION** | | |  |
| Summary of evidence | 24 | Summarize the main findings including the strength of evidence for each main outcome; consider their relevance to key groups (e.g., healthcare providers, users, and policy makers). | 12-13 |
| Limitations | 25 | Discuss limitations at study and outcome level (e.g., risk of bias), and at review-level (e.g., incomplete retrieval of identified research, reporting bias). | 14 |
| Conclusions | 26 | Provide a general interpretation of the results in the context of other evidence, and implications for future research. | 12-13 |
| **FUNDING** | | |  |
| Funding | 27 | Describe sources of funding for the systematic review and other support (e.g., supply of data); role of funders for the systematic review. | 17 |

**Appendix 2:**

**Search strategy for Pubmed (filter is date of publication :1/1/2000-31/10/2021, language is English)** --------------------------------------------------------------------------------

1 Arthroplasty, Replacement, Knee/ mesh term

2 Knee Prosthesis/ mesh term

3 knee replacement. Title/Abstract

4 tkr. Title/Abstract.

5 or/1-4

6 Knee/

7 knee. Title/Abstract

8 or/6-7

9 Arthroplasty/ mesh term

10 Joint Prosthesis/ mesh term

11 (arthroplast* or prosthe* or replace*). Title/Abstract

12 or/9-11

13 8 and 12

14 5 or 13

15 activPal. Title/Abstract

16 function*. Title/Abstract

17 (activity or activities). Title/Abstract

18 (lying or sitting or standing or stepping). Title/Abstract

19 position. Title/Abstract

20 sedentary time. Title/Abstract

21 16 or 17 or 18 or 19 or 20

22 device. Title/Abstract.

23 monitor. Title/Abstract.

24 accelerometer. Title/Abstract.

25 22 or 23 or 24

26 21 and 25

27 15 or 26

28 14 and 27

***************************

**Search strategy for Cochrane (filter is date of publication :1/1/2000-31/10/2021)**

1 Arthroplasty, Replacement, Knee. Mesh term

2 Knee Prosthesis. Mesh term

3 knee replacement. Title, abstract, keyword

4 tkr. Title, abstract, keyword

5 or/1-4

6 Knee. Mesh term

7 knee. Title, abstract, keyword

8 or/6-7

9 Arthroplasty. Mesh term

10 Joint Prosthesis. Mesh term

11 (arthroplast* or prosthe* or replace*). Title, abstract, keyword

12 or/9-11

13 8 and 12

14 5 or 13

15 activPal. Title, abstract, keyword

16 function*. Title, abstract, keyword

17 (activity or activities). Title, abstract, keyword

18 (lying or sitting or standing or stepping). Title, abstract, keyword

19 position. Title, abstract, keyword

20 sedentary time. Title, abstract, keyword

21 16 or 17 or 18 or 19 or 20

22 device. Title, abstract, keyword

23 monitor. Title, abstract, keyword

24 accelerometer. Title, abstract, keyword

25 22 or 23 or 24

26 21 and 25

27 15 or 26

28 14 and 27

***************************

**Search strategy for Embase (filter is date of publication : 2000-2021, and language is English)**

1 Arthroplasty, Replacement, Knee. Emtree exploded

2 Knee Prosthesis. Emtree exploded

3 knee replacement. Title, abstract, keyword

4 tkr. Title, abstract, keyword

5 or/1-4

6 Knee. Emtree exploded

7 knee. Title, abstract, keyword

8 or/6-7

9 Arthroplasty. Emtree exploded

10 Joint Prosthesis. Emtree exploded

11 (arthroplast* or prosthe* or replace*). Title, abstract, keyword

12 or/9-11

13 8 and 12

14 5 or 13

15 activPal. Title, abstract, keyword

16 function*. Title, abstract, keyword

17 (activity or activities). Title, abstract, keyword

18 (lying or sitting or standing or stepping). Title, abstract, keyword

19 position. Title, abstract, keyword

20 sedentary time. Title, abstract, keyword

21 16 or 17 or 18 or 19 or 20

22 device. Title, abstract, keyword

23 monitor. Title, abstract, keyword

24 accelerometer. Title, abstract, keyword

25 22 or 23 or 24

26 21 and 25

27 15 or 26

28 14 and 27

***************************

**Search strategy for Web of science (filter is date of publication : 2000-2021, and language is English)**

1 Arthroplasty, Replacement, Knee. Keyword Plus

2 Knee Prosthesis. Keyword Plus

3 knee replacement. Topic

4 tkr. Topic

5 or/1-4

6 Knee. Keyword Plus

7 knee. Topic

8 or/6-7

9 Arthroplasty. Keyword Plus

10 Joint Prosthesis. Keyword Plus

11 (arthroplast* or prosthe* or replace*). Topic

12 or/9-11

13 8 and 12

14 5 or 13

15 activPal. Topic

16 function*. Topic

17 (activity or activities). Topic

18 (lying or sitting or standing or stepping). Topic

19 position. Topic

20 sedentary time. Topic

21 16 or 17 or 18 or 19 or 20

22 device. Topic

23 monitor. Topic

24 accelerometer. Topic

25 22 or 23 or 24

26 21 and 25

27 15 or 26

28 14 and 27

***************************

**Search strategy for Scopus (filter is date of publication : 2000-2021, and language is English)**

1 Arthroplasty, Replacement, Knee. Keyword

2 Knee Prosthesis. Keyword

3 knee replacement. title, abstract, keyword

4 tkr. title, abstract, keyword

5 or/1-4 Keyword

6 Knee. Keyword Plus

7 knee. title, abstract, keyword

8 or/6-7. Keyword

9 Arthroplasty. Keyword

10 Joint Prosthesis. Keyword

11 (arthroplast* or prosthe* or replace*). title, abstract, keyword

12 or/9-11. Keyword

13 8 and 12. Keyword

14 5 or 13. Keyword

15 activPal. title, abstract, keyword

16 function*. title, abstract, keyword

17 (activity or activities). title, abstract, keyword

18 (lying or sitting or standing or stepping). title, abstract, keyword

19 position. title, abstract, keyword

20 sedentary time. title, abstract, keyword

21 16 or 17 or 18 or 19 or 20. Keyword

22 device. title, abstract, keyword

23 monitor. title, abstract, keyword

24 accelerometer. title, abstract, keyword

25 22 or 23 or 24. Keyword

26 21 and 25. Keyword

27 15 or 26. Keyword

28 14 and 27. Keyword

Appendix 3:

The risk of bais in the included articles based on ROBINS-I tool for before-after study with control group.

|  | Lützner et al., 2014 |
| --- | --- |
| Confounding | Moderate |
| Selection | Moderate |
| Measurements of interventions | High |
| Missing data | Low |
| Measurements of outcomes | High |
| Reporting | Low |
| Overall | High |

Appendix4:

The NIH quality assessment for Before-After (Pre-Post) Studies With No Control Group.

|  | Granat et al., 2020 | Frimpong et al., 2020 |
| --- | --- | --- |
| 1. Was the research question or objective in this paper clearly stated? | Yes | Yes |
| 1. Was the study population clearly specified and defined? | Yes | Yes |
| 1. Were the participants in the study representative of those who would be eligible for the test/service/intervention in the general or clinical population of interest? | Yes | Yes |
| 1. Were all eligible participants that met the prespecified entry criteria enrolled? | Yes | Yes |
| 1. Was the sample size sufficiently large to provide confidence in the findings? | Yes | Yes |
| 1. Was the test/service/intervention clearly described and delivered consistently across the study population? | Yes | Yes |
| 1. Were the outcome measures prespecified, clearly defined, valid, reliable, and assessed consistently across all study participants? | Yes | Yes |
| 1. Were the people assessing the outcomes blinded to the participants' exposures/interventions? | No | No |
| 1. Was the loss to follow-up after baseline 20% or less? Were those lost to follow-up accounted for in the analysis? | Yes | Yes |
| 10. Did the statistical methods examine changes in outcome measures from before to after the intervention? Were statistical tests done that provided p values for the pre-to-post changes? | Yes | Yes |
| 1. Were outcome measures of interest taken multiple times before the intervention and multiple times after the intervention (i.e., did they use an interrupted time-series design)? | No | No |
| 1. If the intervention was conducted at a group level (e.g., a whole hospital, a community, etc.) did the statistical analysis take into account the use of individual-level data to determine effects at the group level? | No | No |
| 12. Summary Quality | Good | Good |
